# The aryl hydrocarbon receptor as a mediator of host-microbiota interplay

**DOI:** 10.1080/19490976.2020.1859812

**Published:** 2020-12-17

**Authors:** Fangcong Dong, Gary H. Perdew

**Affiliations:** Department of Veterinary and Biomedical Sciences and the Center for Molecular Toxicology and Carcinogenesis, The Pennsylvania State University, University Park, PA, USA

**Keywords:** Aryl hydrocarbon receptor, tryptophan, indole, gut microbiota, intestinal homeostasis, immune response, inflammatory disease

## Abstract

Increasing evidence suggests a significant role for microbiota dependent metabolites and co-metabolites, acting as aryl hydrocarbon receptor (AHR) ligands, to facilitate bidirectional communication between the host and the microbiota and thus modulate physiology. Such communication is particularly evident within the gastrointestinal tract. Through binding to or activating the AHR, these metabolites play fundamental roles in various physiological processes and likely contribute to the maintenance of intestinal homeostasis. In recent years, tryptophan metabolites were screened to identify physiologically relevant AHR ligands or activators. The discovery of specific microbiota-derived indole-based metabolites as AHR ligands may provide insight concerning how these metabolites affect interactions between gut microbiota and host intestinal homeostasis and how this relates to chronic GI disease and overall health. A greater understanding of the mechanisms that modulate the production of such metabolites and associated AHR activity may be utilized to effectively treat inflammatory diseases and promote human health. Here, we review microbiota-derived AHR ligands generated from tryptophan that modulate host-gut microbiota interactions and discuss possible intervention strategies for potential therapies in the future.

## Introduction

The human gastrointestinal (GI) tract is a bioreactor with an incredibly diverse and dynamic microbial community referred to as the gut microbiota, which can be considered a functional organ. Over the past decade, tremendous strides have been taken toward understanding the composition and functional capacity of the gut microbiota that resides in the human GI tract^1^. The mechanisms by which these microorganisms contribute to host health have been extensively investigated, and the gut microbiota has been recognized to play a critical role in human health by generating metabolites, which act both locally and systemically to influence human physiology and disease.^2^ Modern analytical methods have revealed that thousands of low molecular weight metabolites are produced that may mediate host-gut microbiota interactions.^3^ Many of these microbiota-dependent metabolites and co-metabolites have been demonstrated to correlate, either positively or negatively with human health or disease. However, a causal mechanism of action has only been identified for a small fraction of the microbiota-dependent metabolites identified thus far. Currently, the most studied categories of metabolites involved in host-gut microbiota interactions, include short-chain fatty acids (SCFA), such as acetate, propionate, and butyrate, produced from the fermentation of dietary fiber, bile acids secreted into the intestinal tract, and tryptophan-derived microbial metabolites.^4,5^ The biological function of these classes of metabolites continues to be refined. Recent evidence has highlighted that many gut derived microbial metabolites modulate aryl hydrocarbon receptor (AHR) activity, which facilitates metabolic communication between the host and gut microbiota.^6^ The mechanisms underpinning such AHR-microbiota communication are multi-factorial, involving modulation of immune tolerance and response,^7^ intestinal homeostasis,^8^ carcinogenesis,^9^ and intestinal barrier integrity.^10^

**Figure 1.**
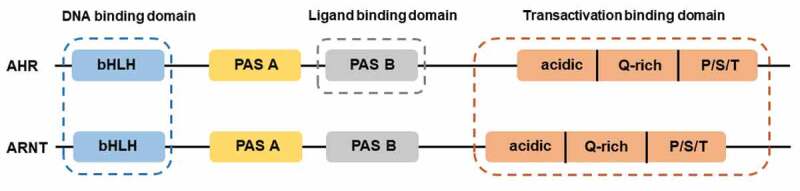
Structures of AHR and ARNT. AHR and ARNT belong to the family of basic helix-loop-helix-Per-ARNT-Sim (bHLH-PAS) proteins. AHR contains a bHLH, a PAS, and transactivation domain. The transactivation domain is divided into three modular transcriptional domains, namely, an acidic region enriched with glutamic and aspartic acid residues, a glutamine-rich region (Q-rich), and a P/S/T region rich in proline/serine/threonine residues. ARNT has the similar structure with AHR. The function of each domain is illustrated above

**Figure 2.**
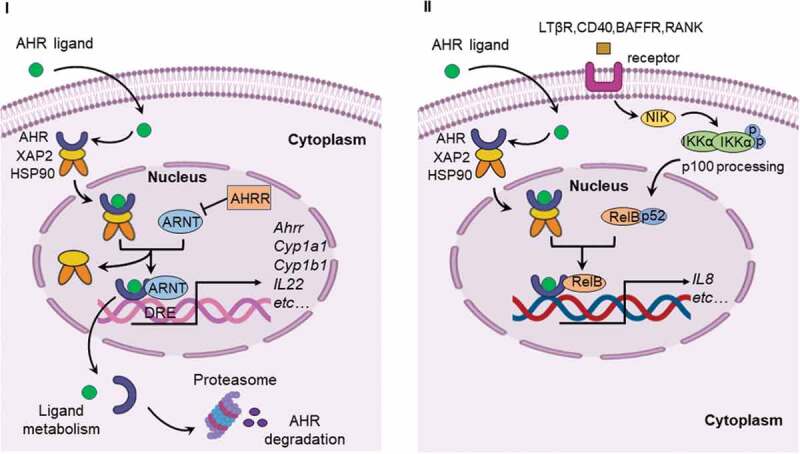
Canonical AHR signaling pathway and alternative AHR/RelB pathway. I. Canonical signaling pathway of AHR. Inactive AHR is retained in the cytoplasm in a multi-protein complex containing chaperone proteins, such as HSP90 and XAP2. AHR translocates into the nucleus after ligand binding and interacts with ARNT to form an AHR/ARNT complex. The AHR/ARNT dimer binds promoter regions containing DRE that regulate expression of numerous target genes, such as *Ahrr, Cyp1a1, Cyp1b1* and *IL22*. II. AHR can also interact with RelB to induce the expression of cytokines and chemokines. The alternative NF-κB pathway is induced by ligands of TNFR superfamily members and is an IKKα-dependent kinase cascade. Activation of this cascade mediates phosphorylation of NIK leading to phosphorylation of IKKα, and subsequent phosphorylation of the p100 NF-κB subunit. This subunit is then cleaved to p52 leading to the formation of the p52/RelB complex. Following translocation of p52/RelB complex to the nucleus, AHR can interact with RelB to form RelB/AhR response element (RelBAHRE) regulating the expression of cytokines and chemokines. AHRR, AHR repressor; ARNT, AHR nuclear translocator; *Cyp1a1*, cytochrome P450 1A1; *Cyp1b1*, cytochrome P450 1B1; DRE, dioxin response element; HSP90, heat shock protein 90; IL22, interleukin 22; XAP2, X-associated protein 2; NF-κB, nuclear factor-κB; LTβR, lymphotoxin β receptor; RANK, receptor activator of NF-κB; BAFFR, B-cell activating factor receptor; CD40, cluster of differentiation 40; NIK, NF-κB-inducing kinase; IKK, IKB kinase; IL8, interleukin 8; RelBAHRE, RelB/AHR response element

AHR, a ligand-activated transcription factor, localized in the cytoplasm in a transcriptionally inactive state as a multimeric complex with two molecules of heat shock protein 90 (HSP90), one molecule of X-associated protein 2 (XAP2, also known as AIP), and one molecule of the HSP90 co-chaperone p23 (P23).^11^ After binding ligand, the activated AHR translocates into the nucleus where it dimerizes with AHR nuclear translocator (ARNT) protein and forms a functional DNA-binding transcription factor. AHR and ARNT, belonging to the family of basic helix-loop-helix-Per-ARNT-Sim (bHLH-PAS) proteins, consisting of the bHLH domain, the PAS domain and the transactivation domain (TAD) where coactivators and corepressors interact (Figure 1).^12^ Ligand binding to AHR occurs within the PAS B domain. However, the PAS B region of ARNT is not able to bind ligands. It is now well established that activated AHR/ARNT complex (AHRC) following ligand binding is capable of recruiting multiple coactivator complexes, such as steroid receptor coactivator 1 (SRC-1),^13^ CREB binding protein (CBP/p300),^14^ nuclear coactivator 2 (NCoA2) and p/CIP,^15^ receptor-interacting protein 140 (RIP140),^16^ coiled-coil coactivator (CoCoA),^17^ GAC63,^18^ NcoA4, and TRIP23,^19,20^ which play significant roles in promoting AHR-responsive gene expression. These coactivator proteins have been reported to be involved in regulation of the transcriptional machinery and remodeling of chromatin structure.^11^ In addition to these classic transcriptional regulators, AHR is recruited by other transcription factors during transcription, such as estrogen receptor-α (ERα),^21^ and nuclear factor-κB (NF-κB).^22,23^ NF-κB containing members of RelB, RelA, c-Rel, p50, and p52,^24^ transcription factors that modulate inflammatory responses. The canonical NF-κB pathway is triggered through an induction of toll-like receptor (TLR) signaling by bacterial products such as lipopolysaccharide (LPS) or cytokine production such as tumor necrosis factor α (TNFα) and IL1, leading to activation of RelA/p50 heterodimers mediating expression of proinflammatory and cell survival genes.^25^ The interaction between AHR and NF-κB is bidirectional. AHR agonists has been shown to suppress NF-κB mediated-gene expression.^26^ Likewise, RelA/p50 also repressed AHR transcriptional activity.^27^ Interestingly, a direct physical interaction between AHR and RelB was also observed.^28^ Alternative NF-κB pathway is activated by ligands of a subset of the tumor necrosis factor receptor (TNFR) superfamily members, such as lymphotoxin β receptor (LTβR), B-cell activating factor receptor (BAFFR), cluster of differentiation 40 (CD40), or receptor activator of NF-κB (RANK). The release of NF-κB-inducing kinase (NIK) activates and cooperates with IκB Kinase α (IKKα) to induce p100 phosphorylation (Figure 2).^29^ RelB/p52 dimer is produced and translocates into nucleus through the processing of p100.^30^ RelB/AHR binding element (RelBAHRE) is formed by the interaction between AHR and RelB, regulating the expression of cytokines and chemokines.^31^ Several reports have shown that multiple inflammatory genes, including chemokines such as monocyte attracting protein (MCP), interleukin 8 (IL8), and CC-chemokine ligand 1 (CCL1), are regulated in an AHR-dependent manner.^32–34^ Upon exposure to AHR ligands, the AHR/ARNT heterodimer binding to dioxin response elements (DRE) alters numerous AHR target genes, such as cytochrome P450 (CYP) *Cyp1a1* and *Cyp1b1*, and *Ahrr* (Figure 2).^35^ CYP1A1 and CYP1B1 encoded by *Cyp1a1* and *Cyp1b1* respectively, belong to the cytochrome P450 superfamily of enzymes and have been shown to have a profound impact on the metabolism of xenobiotics.^36^ Expression of the target gene *Ahrr* is enhanced by activation of AHR. Interestingly, the generation of AHR repressor (AHRR) serves as an important negative feedback mechanism to limit the availability of ARNT to form AHR/ARNT dimers.^37^ Additionally, targeted protein degradation of the AHR downregulates AHR signaling. After being exported out of the nucleus, AHR is rapidly degraded in the cytoplasmic compartment by the proteasome.^38^ Transactivation activity of AHR is also repressed by post-translational modifications such as its SUMOylation.^39^ AHR was initially discovered as a primary mediator of the toxic effects arising from exposure to man-made environmental contaminants, such as polycyclic or halogenated aromatic hydrocarbons (PAHs or HAHs). For example, AHR was initially identified as the high-affinity receptor for the environmental toxicant 2,3,7,8-tetrachlorodibenzo-*p*-dioxin (TCDD), a type of HAH.^40^ The role of the AHR as a xenobiotic sensor responsible for mediating such toxicity and tumor promotion has dominated research on this receptor for decades. Consequently, the underlying physiological ‘raison d’êtra’ of AHR is only now being appreciated.

As stated previously, activation of AHR has immunomodulatory effects through interaction with NF-κB. Compelling evidence has accumulated supporting a role for the AHR in regulating adaptive immune responses relevant to the pathogenesis of diseases, such as inflammatory bowel disease (IBD), multiple sclerosis (MS), rheumatoid arthritis (RA), cancer, and obesity.^7,41–44^ These observations have led to the hypothesis that the AHR participates in innate immune responses to microbial invasion of barrier tissues. The combination of a TLR ligand (e.g. LPS) and a microbially generated metabolite that activates the AHR could lead to an enhanced inflammatory response.

Thus, the study of AHR regulation and function is likely to reveal unknown biological processes highly related to the development of inflammatory disorders and may realize the potential of an AHR as a novel therapeutic intervention for clinical use. There are a number of reviews published that focus on the identification of exogenous and endogenous AHR ligands.^45,46^ While here, we review the most recent insights related to microbial AHR ligands produced from host-gut microbiota metabolism with a focus on tryptophan and indole metabolites. Also, we discuss the role of these endogenous or microbially generated metabolites via ligand-dependent activation of AHR to regulate inflammation in multiple chronic diseases.

## AHR ligands produced by host-gut microbiota interaction

**Figure 3.**
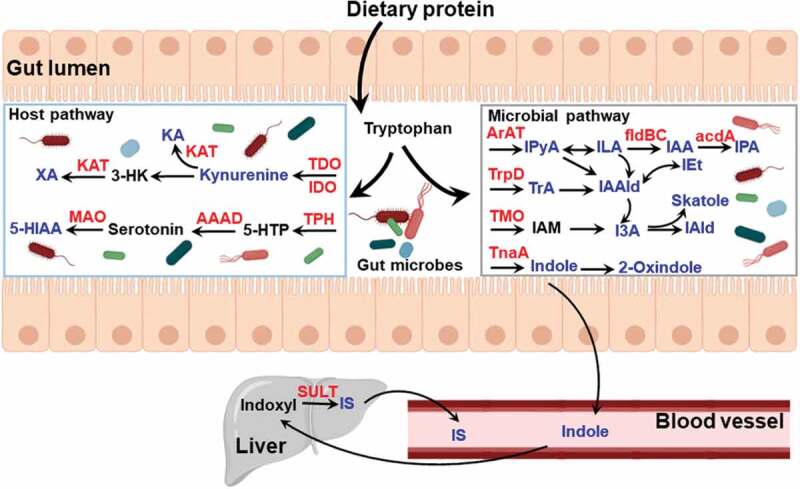
Summary of tryptophan and indole metabolites generated by host and gut microbiota metabolism. Tryptophan is acquired from dietary protein digested in the small intestine and converted to various catabolites. The key metabolites (in blue) have been identified to be AHR ligands and enzymes involved in metabolism are represented in red. AAAD, aromatic amino acid decarboxylase; TDO, tryptophan 2,3-dioxygenase; IDO, indoleamine 2,3-dioxygenase; KA, kynurenic acid; KAT, kynurenine aminotransferase; MAO, monoamine oxidase; TPH, tryptophan hydroxylase; 5-HIAA, 5-hydroxyindole-3-acetic acid; 3-HK, 3-hydroxykynurenine; 5-HTP, 5-hydroxytryptophan; ArAT, aromatic amino acid transaminase; fldBC, phenyllactate dehydratase; IAA, indole acrylic acid; I3A, indole-3-acetic acid; IAld, indole-3-aldehyde; IAAld, indole-3-acetaldehyde; IAM, indole-3-acetamide; ILA, indole-3-lactic acid; IEt, indole-3-ethanol; IPyA, indole-3-pyruvate; IPA, indolic-3-propionic acid; TMO, tryptophan-2-monooxygenase; TnaA, tryptophanase; TrpD, tryptophan decarboxylase; IS, indoxyl sulfate; TrA, tryptamine; SULT, sulfotransferase

Ligand-dependent activation of the AHR is mediated by diverse sources, including environmental pollutants, dietary components, and endogenous metabolites. Endogenous mechanisms of AHR activation, independent of xenobiotics, were recognized by the development of *Ahr^−/-^* mice, which revealed developmental defects in the immune system.^47,48^ Increasingly, evidence indicates that the AHR has a wider role than previously recognized, mediating diverse biological processes. Thus, it is critical to further characterize endogenous molecules that influence receptor activity and how that informs us about the AHR in physiological processes. Much interest has focused on a search for putative endogenous AHR ligands. In recent years, many studies have shown that gut microbiota residing in the GI tract provides a rich source of pseudo-endogenous AHR ligands. Interestingly, endogenous AHR ligands have largely been identified as metabolites of tryptophan and indole metabolism (Figure 3). Furthermore, production of pseudo-endogenous AHR ligands in the GI tract appears to be dependent on the types of bacteria present.^49,50^ However, although tryptophan itself neither exhibits AHR-binding capacity nor the ability to induce AHR activity, a number of endogenous metabolites of tryptophan are recognized as AHR ligands, including tryptamine (TrA), indole, 2-oxindole, 3-methylindole (skatole), indoxyl sulfate (IS), indole-3-acetic acid (I3A), indole-3-propionic acid (IPA), indole-3-pyruvate (IPyA), indole acrylic acid (IAA), Indole-3-aldehyde (IAld), indole-3-lactic acid (ILA), 5-hydroxyindole-3-acetic acid (5-HIAA), kynurenine, kynurenic acid (KA), and xanthurenic acid (XA).^46^ The majority of previous studies have focused on the ability of endogenous metabolites to activate AHR, largely without regard to whether the concentration present in the intestinal tract is at a concentration that is physiologically relevant in terms of activating the AHR. Thus, an understanding of key endogenous AHR ligands within the GI tract has not been properly defined, especially in non-pathological conditions. Such knowledge could be beneficial in investigating the physiological roles of the AHR in host-gut microbiota interactions. In a recent study, a panel of tryptophan metabolites capable of activating AHR were quantified in cecal contents from mice and human stool under physiologically “normal” conditions.^51^ Importantly, indole, 2-oxindole, I3A, and KA derived from tryptophan metabolism pathways by gut microbiota were recognized as the primary sources of AHR activation within the GI tract based on concentrations present and AHR activation potential. This is the first study to identify the importance of microbially produced 2-oxindole and KA to AHR activation within the gut. In addition, these tryptophan metabolites exhibit far greater *Cyp1a1* activation potency for the human AHR when compared to the mouse AHR. These results need to be taken into account when considering the limitations of the mouse model and its extrapolation to humans. Interestingly, the human AHR has a key amino acid residue, valine 381, in the ligand-binding pocket that is an alanine in other primates; this results in the human AHR having lower affinity for exogenous ligands, such as PAHs.^52^ However, the amino acid change does not affect the affinity for indole, which suggests that the ability of endogenous tryptophan metabolites to activate the AHR is conserved across all primates.

## AHR ligands and host metabolism in tryptophan catabolism

Tryptophan is an essential amino acid that can only be acquired through dietary protein. Dietary tryptophan is liberated into the small intestine through proteolytic digestion. The majority of free tryptophan is absorbed through intestinal epithelium, principally along the jejunal-ileal axis, and is utilized for protein synthesis by the host; while ~10-20% of tryptophan is further metabolized, the kynurenine and serotonin pathways or by gut microbes (Figure 3).^53^ Generation of endogenous AHR ligands, such as kynurenine and KA, is largely achieved through tryptophan metabolism in host cells via the enzymes indoleamine 2,3-dioxygenase (IDO) and tryptophan 2,3-dioxygenase (TDO).^54^ Kynurenine is a low-affinity AHR ligand that has been linked to regulatory T (Treg) cell functional maturation and suppression of inflammatory cytokine production in dendritic cells (DCs), altering the progression of inflammation leading to immune dysfunction.^55^ KA, generated by transamination of kynurenine via kynurenine aminotransferases (KAT) activity, is found to be a relatively potent ligand for human AHR and co-mediates the induction of IL6 expression coupled with inflammatory signaling.^56^ Also, this study found that XA generated by transamination of the kynurenine metabolite 3-hydroxykynurenine (3-HK), activated AHR in a DRE-driven luciferase reporter in human hepatoma cells. Serotonin (5-hydroxytryptamine, 5-HT) known as a brain neurotransmitter, is largely produced in the gut from the amino acid tryptophan via hydroxylation and decarboxylation. Over 90% of serotonin in the body is synthesized by colonic enterochromaffin cells (ECs).^57^ Gut-derived serotonin is regulated by the gut microbiota, particularly spore-forming bacteria.^58^ Yano *et al*. showed that 60% less serotonin were produced in the ECs from germ-free mice compared to specific pathogen-free mice. In a recent study, Manzella *et al*. found serotonin is an endogenous activator of AHR.^59^ While it is not a ligand for AHR, serotonin acting as a CYP1A1 substrate, interferes with metabolic clearance of AHR ligands through inhibition of CYP1A1 activity leading to a decrease in ligand degradation and promotion of AHR activation in intestinal epithelial cells.^60,61^ Clearly, whether other microbial metabolites are capable of modulating AHR activity through inhibition of CYP1A1 should be explored. In a recent study, a novel AHR ligand of 5-HIAA derived from serotonin by monoamine oxidase (MAO) activity was discovered to be a transcriptional marker for regulatory B cells function via AHR activation.^42^

## AHR ligands and yeast infections

Providing vital protection from physical or chemical harm and from infection, the skin serves as the barrier against the environment. A significant amount of AHR ligands can be produced by microorganisms of the skin microbiome, e.g., Malassezia. Malassezia yeast are commensal microbes that, under certain host pathological conditions, can result in disease. Culturing a number of specific strains of Malassezia in tryptophan-rich media results in the production of a number of tryptophan metabolites with potent AHR agonist activity, such as indole[3,2b]carbazole (ICZ), 6-formylindolo[3,2b]carbazole (FICZ), and malassezin.^62,63^ Extracts isolated from skin scale from patients with Malassezia-associated diseases revealed an increase of 10–100-fold increase in AHR activation potential compared to control skin extracts. A number of potent AHR ligands were identified in these extracts that are seen in Malassezia yeast cultures. Such observations were among the first to establish a direct relationship between the microbiota, its associated metabolites, AHR, and pathology. It would be expected that potent activation of the AHR in the host epithelium, coupled with the presence of the yeast, would result in a heightened inflammatory response. Extra-mammary Paget’s disease (EMPD) is a skin cancer and the possible role of Malassezia-derived AHR ligands in the progression of the disease has been investigated.^64^ In lesioned tissue, EMPD keratinocytes expression of the AHR target genes CYP1A1 and CCL20 is enhanced. These results are consistent with the assertion that AHR activation participates in inflammatory signaling in human disease. In fact, a previous study by Zelante *et al*. has revealed the activation of AHR by tryptophan metabolites provides antifungal resistance to *Candida albicans* via interleukin-22 and mucosal protection from inflammation.^50^ In another yeast-mediated disease, oropharyngeal candidiasis, which is most often associated with *Candida albicans* infection of the oral epithelium in AIDS patients, the AHR may be involved in the progression of the disease.^65^ Kynurenine activated AHR in oral epithelial cells increases activation of Src family kinases that enhances fungal endocytosis. These studies reinforce the concept that microbes that produce AHR ligands can modulate disease status and offers the potential of therapeutic intervention with AHR modulators.

## AHR ligands and gut microbiota in tryptophan catabolism

A number of studies have revealed the microbiota as a driving force mediating the degradation of tryptophan in the large intestine. Interestingly, a number of endogenous AHR ligands are produced by metabolic activity of microbiota that inhabit the GI tract. For instance, TrA, produced by tryptophan decarboxylases (TrpDs) from *C. sporogenes* and *Ruminococcus gnavus*,^66^ exhibit AHR-dependent anti-inflammatory activities in Caco2 intestinal cells.^67^ In addition, TrA is the precursor of IAAld, which also manifests as an AHR ligand.^43^ Indole-3-ethanol (IEt), known as tryptophol, is formed from IAAld by alcohol dehydrogenase. A recent study determined that IEt, IPyA and IAld derived from tryptophan by gut microbiota could regulate gut barrier function via AHR activation.^68^ IPyA, a transamination product of dietary tryptophan by microbiota, such as Clostridium sporogenes,^69^ is a precursor of several microbiota-derived AHR agonists, such as I3A, IAld, and IAAld, and can form various AHR agonists in the reaction of aqueous solution.^70^ Several intestinal bacteria, including *Bacteroides fragilis, Bacteroides thetaiotaomicron* and *Citrobacter sp.^71^* and clostridia,^72^ *Clostridioides difficile,^73^ Clostridium sticklandii, Clostridium lituseburense, Clostridium subterminale* and *Clostridium putrefaciens*, can produce I3A from tryptophan. One study revealed that I3A regulated proinflammatory and oxidative effects in endothelial cells, and activated a signaling pathway related to AHR, resulting in cyclooxygenase-2 (COX2) expression.^74^ Levels of I3A in serum have been associated with high rates of mortality and cardiovascular events in patients with chronic kidney disease (CKD), thus I3A is suggested to be a predictor of mortality and major cardiovascular events in CKD.^74^ Also, a study reported that I3A, which was reduced in mice on a high-fat diet versus a low-fat diet, attenuated cytokine-mediated lipogenesis in hepatocytes, playing a potential protective role in lipid metabolism.^43^ Some strains of *Lactobacillus reuteri* and *Lactobacillus johnsonii* can induce the generation of IAld from tryptophan via several pathways (Figure 3).^50^ In mice, AHR activation by IAld induced IL22 production resulting in enhanced restoration of mucosal immune homeostasis.^50^ 3-Methylindole (skatole) is formed by the decarboxylation of I3A by *Lactobacillus* sp. in the large intestine of humans.^75^ In a study by Hubbard *et al*., in primary human bronchial epithelial and Caco2 cells, skatole can activate AHR at physiologically attainable concentrations to promote the expression of AHR target genes, such as CYP1A1. In addition, skatole acts as a competitive antagonist of TCDD-mediated AHR activation, indicating that skatole is a weak AHR agonist.^76^ Through aromatic amino acid aminotransferase (ArAT) and indolelactic acid dehydrogenase (ILDH) activity,^77,78^ tryptophan can be converted to ILA. It has been reported that ILA could be produced by *Bifidobacterium spp* and *Clostridium Sporogenes.^69^^,^^79^* Meng *et al*. reported ILA suppressed the transcription of inflammatory cytokine IL8 by interacting with AHR activation.^80^ Recently, a study using untargeted metabolomics analyses found two tryptophan microbial metabolites, IPA and IAA, are produced by *Peptostreptococcus. russellii* and *Peptostreptococcus. stomatis* in a phenyllactate dehydratase *fld*ABC-dependent manner; In addition, metagenomic analysis of stool samples from IBD patients revealed that production of IAA is associated with anti-inflammatory activity.^81^

Indole metabolism has also been revealed as a rich reservoir of AHR ligands. Indole is synthesized from tryptophan by microbial tryptophanase (*TnaA*) activity, first recognized in the late 1800s. Many bacterial species encoding the *TnaA* gene can produce indole, consisting of Gram-positive bacteria such as, *Bacillus alvei^82^* and *Clostridium tetani,^72^* and Gram-negative bacteria such as *E. coli,^83^ Bacteroides thetaiotaomicron,^84^ Pasturella multocida^85^* and *Vibrio cholerae* (previous *Asiatic cholera*).^86^ Consequently, indole is present within the intestinal lumen and human feces at micromolar concentrations.^87^ Oxidation of indole to 2-oxindole is putatively involved in the pathophysiology of hepatic encephalopathy.^88^ A study by Hubbard *et al*. suggests indole and 2-oxindole at physiological concentrations can activate the human AHR within the GI tract.^89^ Oxidation and sulfation of indole by hepatic oxidases, including cytochrome P450 2E1 (CYP2E1) and sulfotransferase 1A1 (SULT1A1), generate the systemic AHR ligand IS.^90,91^ IS is typically subject to nephrotic elimination but reaches high serum levels during kidney failure and chronic kidney disease and manifests as an uremic toxin. As a potent endogenous AHR agonist, IS selectively activates the human AHR at nanomolar concentrations in primary human hepatocytes and regulates transcription of multiple genes.^92^ Studies have revealed that the formation of serum levels of IS in mice is dependent upon the presence of gut flora and suggests a linkage between the gut microbiota and kidney disease.^93,94^ In CKD patients, serum concentrations of IS are greater than 50-fold higher than those in healthy subjects.^95^ These studies lead to the conclusion that the bacterial composition of the microbial community, the amount of bacterial metabolic activity, and the amount of available tryptophan in the gut will dictate the level of AHR ligands generated by the microbiota. However, the appropriate level of these AHR ligands to maintain optimal health will need to be further explored.

## AHR-dependent regulation in health and diseases via tryptophan and indole metabolites

In the last few decades, it has become apparent that the gut microbiota is an important deterrent or contributor to many noninfectious disease states. In particular, microbial metabolites have been shown to facilitate the interaction between host and gut microbiota involved in systemic homeostasis across the so-called microbiota-gut-brain and microbiota-gut-liver axes.^96,97^ Although the mechanisms of microbial metabolites mediating signal between host and gut microbiota remain poorly understood, recent studies revealed AHR to be a key regulator in such interaction between host and gut microbiota. Next, we discuss the microbial metabolites that activate the AHR regulating crosstalk between the host and gut microbiota under pathological conditions.

## AHR and inflammatory bowel disease

The etiology of IBD consisting of Crohn’s disease (CD) and ulcerative colitis (UC) remains incompletely understood,^98^ but is believed to be multi-factorial, involving genetic predisposition, lifestyle, diet, and a strong microbiota component.^99,100^ In a recent study, the association between serum levels of tryptophan and its metabolites in patients with IBD, and clinical and serologic features was systematically evaluated.^101^ Subsequently, reduced levels of tryptophan were observed in the serum from IBD patients and there was an inverse association between disease activity and serum levels of tryptophan. This negative correlation could also be observed in dextran sulfate sodium-induced colitis in mice.^102^ Furthermore, the microbiota from mice deficient in caspase recruitment domain 9 (CARD9), a susceptibility gene for colitis,^103^ failed to produce AHR ligands metabolized from tryptophan.^104^ Lamas *et al*. found that the subsequent reduction in AHR activity was associated with reduced IL22 expression and enhanced susceptibility to colitis. Interestingly, not only tryptophan but also microbiota-derived metabolites from tryptophan and indole, could modulate the severity of IBD. Diminished I3A levels and AHR activation are also observed in fecal samples from patients with IBD compared with healthy individuals.^104^ In addition, in experimental colitis models IPyA is shown to be a potent AHR agonist and exhibits significant anti-inflammatory effects, through increased abundance of colonic IL10 positive T cells combined with a reduction in pro-inflammatory Th1 cells.^105^ Several studies have revealed that microbiota-dependent tryptophan metabolites, such as indole, serve an important role in maintaining intestinal homeostasis through AHR activation.^106,107^ These observations suggest tryptophan metabolites and AHR activation potential are involved in the etiology or progression of IBD, thus providing a promising target of therapeutic or nutritional intervention for IBD treatment.

## AHR and central nervous system

In recent years, there has been significant interest in the gut–brain axis, and data suggest that AHR agonists provided by the interaction of the gut microbiota and host metabolism, can cross the blood-brain barrier and contribute to control of central nervous system (CNS) function. Emerging evidence suggests that dysregulation of tryptophan metabolism by the kynurenine pathway is associated with neurodegenerative and other neurological disorders, along with psychiatric diseases.^108^ The production of downstream products of the kynurenine pathway is dependent on expression of IDO and TDO. Considering the expression of IDO/TDO is regulated by activation of AHR;^109,110^ tryptophan catabolites via kynurenine pathway are involved in immune, metabolic and neural communication mechanisms in gut-brain axis, which is controlled by AHR activation.^111^ In addition, certain microbiota that produce indole and indole derivatives from dietary tryptophan can also control astrocyte activity to influence CNS inflammation.^112^ Another study reported AHR agonists in serum are altered during different stages of multiple sclerosis,^113^ the most common neurological autoimmune disease of CNS. In this cohort study, reduced AHR activation in patients with relapsing-remitting MS was observed when compared to healthy controls. Also, a decrease of AHR transcriptional activity was detected in patients with MS in remission compared to those with active inflammation. Interestingly, there was no difference between patients with benign MS and controls, while an increase in agonist-mediated AHR activity is observed in patients with acute inflammation. These results suggest that deficits in AHR agonists provided by interaction between host metabolism and gut microbiota may contribute to the pathogenesis of MS. These AHR agonists represent a potential biomarker to predict disease stages of MS, including relapse frequency and disability progression, which is often difficult to predict even with a combination of clinical criteria, magnetic resonance imaging, and cerebrospinal fluid findings.

## AHR and rheumatoid arthritis

Millions of people worldwide suffer from the autoimmune disorder of RA and its mortality is increasing. Although the mechanisms driving RA progression are not well understood,^114^ recent data implicate the gut microbiota in the etiology of RA.^115^ In a recent study by Rosser *et al*., RA patients exhibited reduced levels of the microbiota-derived SCFA butyrate compared to healthy controls; and butyrate levels showed a significant positive correlation with the frequency of total CD19+ CD24^hi^CD38^hi^B cells and with IL10^+^B cells.^42^ SCFAs generated by gut microbiota are known to play a vital role in the homeostasis of intestinal epithelium, including providing nutrients for intestinal epithelial cells, supporting barrier function and regulating the production of cytokines and chemokines.^2^ Notably, studies of activation of the AHR by TCDD in mice have highlighted its role for differentiation and function of adaptive immune cells, such as T cells and DC cells.^116–118^ Consistent with AHR activation inducing immunosuppression, Piper *et al*. observed that AHR played a critical role in inducing regulatory B cells differentiation and IL10 transcription to attenuate autoimmunity and inflammation.^119^ Building on these findings, a significant increase in microbiota-induced AHR ligand 5-hydroxyindole-3-acetic acid (5-HIAA), metabolized from serotonin, was discovered to attenuate arthritis with butyrate supplementation by promoting IL10 transcription in B cells in an AHR-dependent manner.^42^

## AHR role in obesity and gut/liver axis

Obesity is a global health problem that is exacerbated by western diets high in caloric content, fat and carbohydrate and often characterized by the low consumption of fruit and vegetables. Along with obesity, fatty liver and increased visceral fat can lead to an elevation in inflammatory signaling. Accumulating evidence reveals that the AHR plays a key role in the crosstalk between obesity and gut microbiota. The supplementation of the diet in mice with an AHR ligand (e.g. FICZ) or a *Lactobacillus* strain that produces AHR ligand(s) is capable of improving metabolic syndrome factors, such as hepatic triglyceride levels, fasting glucose levels, and insulin in *ob/ob* mice.^120^ Importantly, in humans, a high body mass index correlated with a reduced level of AHR active tryptophan metabolites and overall AHR activation potential in fecal extracts. A similar level of AHR activity in stool was shown in fecal microbiota transplantation experiment. Germ-free mice colonized with fecal microbiota of high-fat diet-fed mice resulted in a reduction of AHR activity compared with mice colonized with microbiota of conventional diet-fed microbiota. These findings indicate a decrease of microbiota-derived AHR ligands was associated with a shift in composition of gut microbiota in obese humans. In fact, a lower abundance of Bacteroidetes was observed in the obese mice compared to lean mice.^121^ Another possible explanation that can, at least in part, provide a mechanism for this observation is the ability of AHR activation in the GI tract to induce IL22 production and its subsequent ability to attenuate adverse metabolic parameters in *ob/ob* mice.^122^ This observation, coupled with results demonstrating that mice deficient in IL22 receptor expression fed a high-fat diet are susceptible to the development of metabolic dysfunction, would suggest that AHR activation in the gut has a protective effect against obesity. Importantly, AHR activation provides protection for the stem cell niche in the intestinal epithelium through restricting cell proliferation and influencing differentiation and enhances overall barrier function.^123^

A different story emerges from studies designed to examine the influence of AHR activation in the liver on obesity. Transgenic mouse models that express a constitutively activated AHR in the liver have revealed that elevated expression of the CD36/fatty acid translocase results in a fatty liver phenotype through the increased uptake of fatty acids.^124^ The administration of 3-methyl cholanthrene, a potent AHR ligand, in C57BL6/J mice results in an increase in both liver triglycerides and CD36 expression.^125^ This would suggest that high-level AHR activation can lead to fatty liver. Another possible mechanism is the ability of CYP1B1 enzyme to influence fatty acid metabolism and prevent obesity, supported by results obtained in *Cyp1b1^−/-^* mice.^126^ The loss of *Cyp1b1* expression in mice is correlated with suppression of high-fat diet (HFD)-induced obesity via decreased expression of stearoyl-CoA desaturase 1 (SCD1),^127^ the rate-limiting enzyme synthesizing monounsaturated fatty acids from saturated fatty acids.^128^ Moreover, expression of PPARγ and target genes regulated by PPARα are also reduced in *Cyp1b1* knockout mouse.^129^ Activation of PPARγ promotes induction of adipocytes from preadipocytes and storage of triglyceride, while genes stimulated by PPARα plays a critical role in fatty acid transport and mitochondrial fatty acid β-oxidation.^130^ Taken together, these results suggest the involvement of AHR in regulation of CYP1B1 expression, which promotes fatty acid synthesis, and inhibition of CYP1B1 could be a potential target for clinic treatment of obesity. Indeed, dietary exposure to AHR antagonist α-naphthoflavone is capable of inhibiting fatty liver and obesity in mice fed a western diet.^131,132^

## Therapeutic potential of AHR in clinical applications

**Figure 4.**
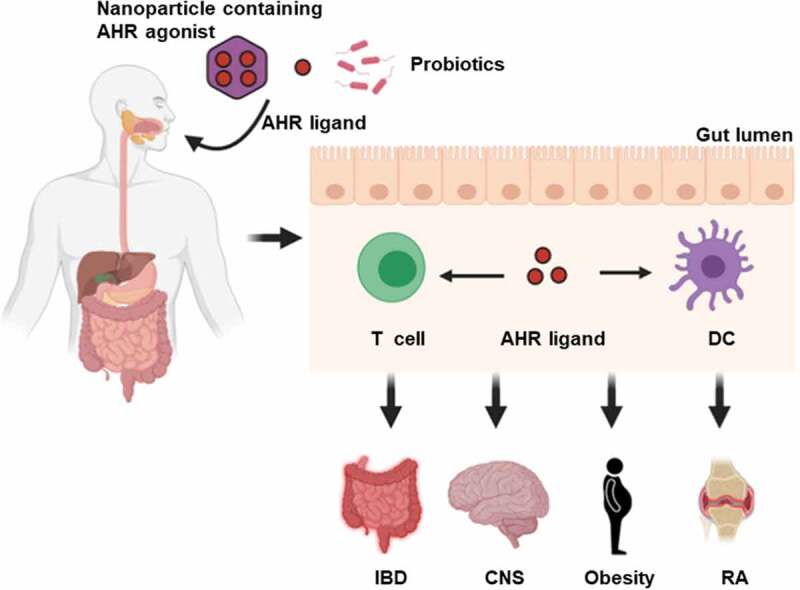
AHR as a therapeutic target. Novel strategies such as oral administration of nanoparticle-mediated cell-type-specific delivery of AHR agonist, AHR ligands and specific probiotics may serve to influence autoimmune inflammatory response. DC, dendritic cell; IBD, inflammatory bowel disease; CNS, central nervous system; RA, rheumatoid arthritis

Recent technical progress in the field of global untargeted metabolomics, which focuses on identifying low molecular weight metabolites that impact the host, have facilitated the screening of microbial metabolites that may induce AHR activity and subsequently influence host-gut microbiota homeostasis. Such analyses performed on cecal contents from conventional and germ-free mice with subsequent activity assays have identified 2,8-dihydroxyquinoline (2,8-DHQ) as a microbial-derived AHR ligand.^133^ In addition, accumulating evidence suggests that AHR plays a vital role in the regulation of inflammatory signaling leading to changes in the abundance and function of various immune cells involved in both the innate and adaptive immune systems, such as Th17, Treg and DC.^134^ Taken together, progress in the identification and function of microbiota-derived AHR ligands may lead to novel therapeutic options for multiple inflammatory diseases. Indeed, the therapeutic modulation of the gut microbiota and consequently microbial AHR ligands is being investigated for improving autoimmune diseases, such as IBD, CNS disease, and metabolic syndrome (Figure 4).^112,135^ For example, probiotics containing *L. reuteri*, which have been demonstrated to generate AHR agonists, have been successfully utilized to alleviate inflammation.^136^ Not only the use of AHR agonist-producing probiotics, but also direct oral administration of microbial AHR agonists, such as IPA, indole, and IPyA, have been shown to reduce experimental intestinal inflammation in an experimental colitis model.^105,137,138^ A recent study demonstrated that a diet supplemented with the tryptophan metabolites indole, IS, IPA and IAld limited CNS inflammation in antibiotic-treated mice.^112^ Furthermore, in order to minimize off-target AHR effects in non-targeted tissues, nanoparticles have been used for the targeted delivery of AHR agonists to the desired tissues and cells.^139^ Current research has shown that, antigen delivered by nanoparticle can be used to promote protective immunity against microorganisms and cancer.^134^

## Conclusions

Numerous studies now support the conclusion that the AHR plays an important role in maintaining gut homeostasis both under homeostatic conditions and during chemical or bacterial challenge. However, there is a need to further expand our understanding of the role for the AHR in multiple cell types within the intestinal tract, including various immune cell types, in the gut epithelium, in the underlying lamina propria, and in the enteric nervous system. This information will be critical to understanding what level of pseudo-endogenous ligands (e.g. tryptophan metabolites) or dietary ligands (e.g. indolo[3,2*b*]carbazole) is optimal in maintaining healthy gut health. Whether these microbial or dietary AHR ligands could be used for therapeutic purposes also warrant further investigation. This concept is supported by the observation that indole-3-carbinol can attenuate the impact of *C. difficile* infection in mice.^140^ Yet another area of investigation involves understanding the impact of interspecies difference in AHR activation. In particular, it is important to note that there are several important structural differences in the human versus mouse AHR, thus raising concern about the use of the mouse models. Nevertheless, most studies on microbial AHR ligand intervention have been evaluated in mouse model. A number of studies establish that the affinity of the AHR for TCDD in mice is 10-fold higher than in humans.^141^ In contrast, IS is a more potent ligand for the human AHR and has a 500-fold higher potency for human versus the mouse AHR.^92^ In addition, another study revealed that different levels of species-dependent AHR activation were observed for the indole derivatives; indole, 2-oxindole and skatole, which potently activate the human AHR in a dose-dependent manner, whereas only low concentrations of IPA could induce activity.^89^ Yet another area to explore is the exact function of each AHR ligand in the different intestinal segments and cell types. Importantly, these microbial AHR ligands from tryptophan and indole metabolism exhibited markedly different concentrations in the luminal contents. For example, Karlin *et al*. identified the quantity of indole in human feces as a major microbial metabolite, at concentration of ~58.0 µg/g.^87,142^ Skatole in human fecal matter varies considerably, with an average quantity of 34.5 µg/g.^87^ A recent study reported 2,8-DHQ, a human AHR-selective ligand, was quantified in human feces with the concentrations between 0 and 3.4 pmol/mg.^133^ These observations bring up the question whether the microbiomes in individual humans differ in their capacity to produce tryptophan metabolites. Thus, a more comprehensive understanding of the complex effects of microbiota derived AHR ligands, capable of modulating overall gut health and subsequent resistance to disease, is needed. Given the important role of AHR as a mediator in host-gut microbiota communication, this review highlights the utility of the AHR as a promising therapeutic target in human disease, which can be altered through changes in the microbiota, modifying diet, or the administration of drugs.
